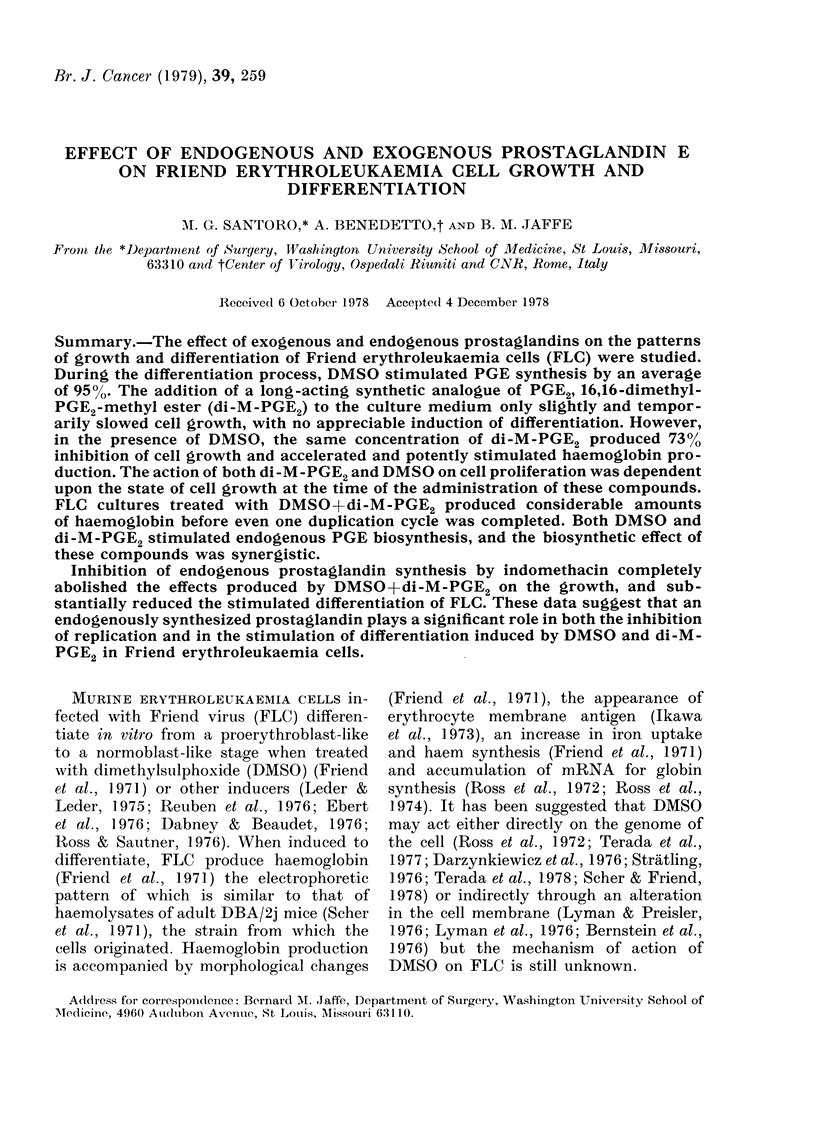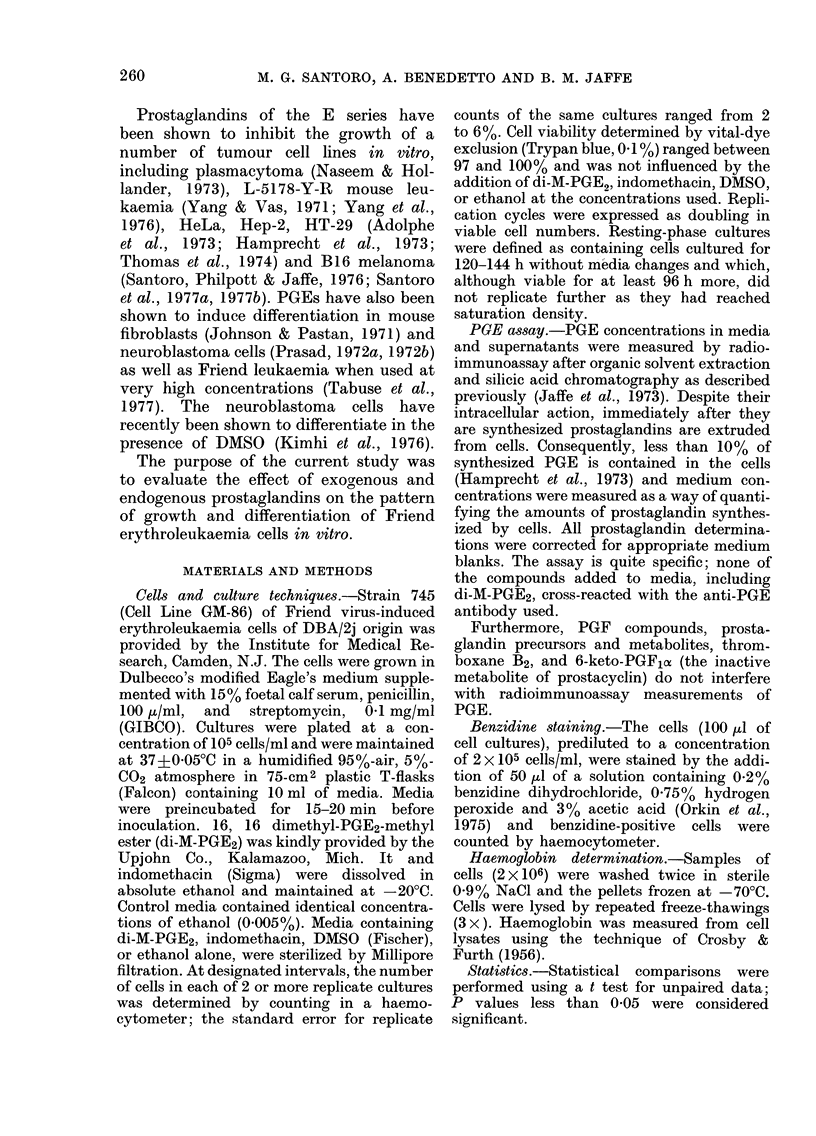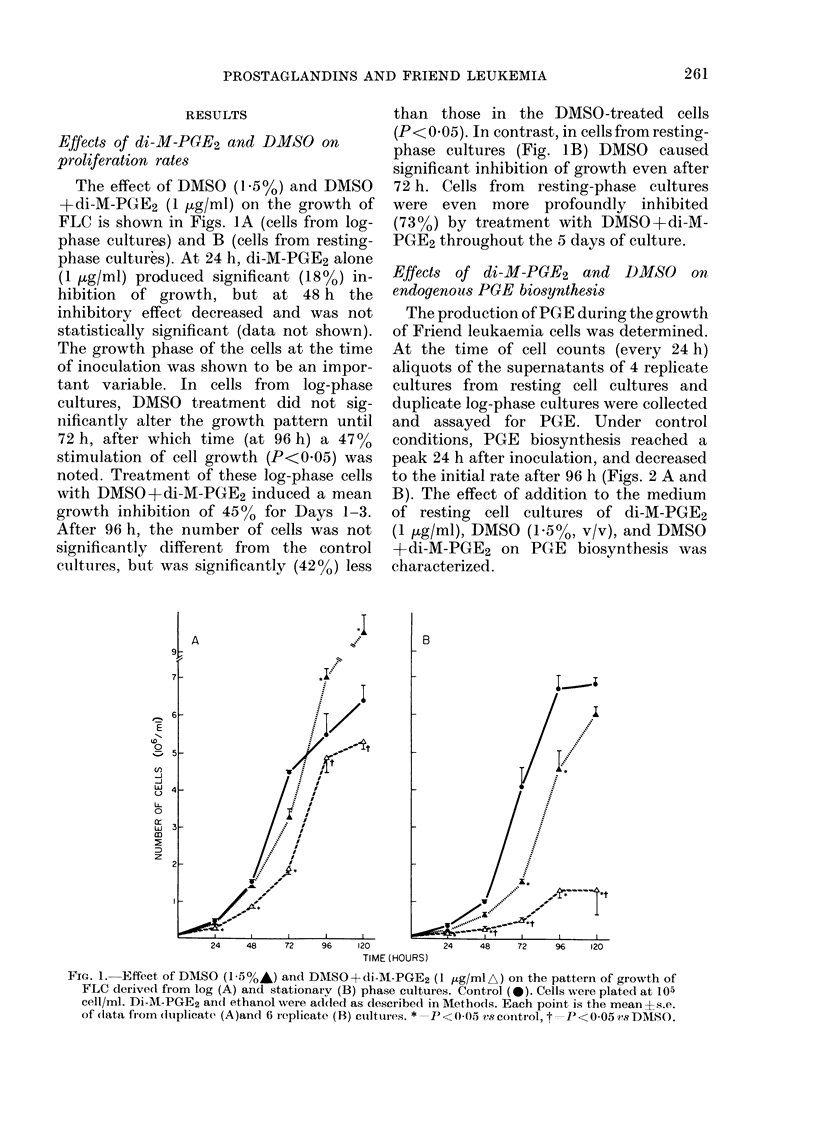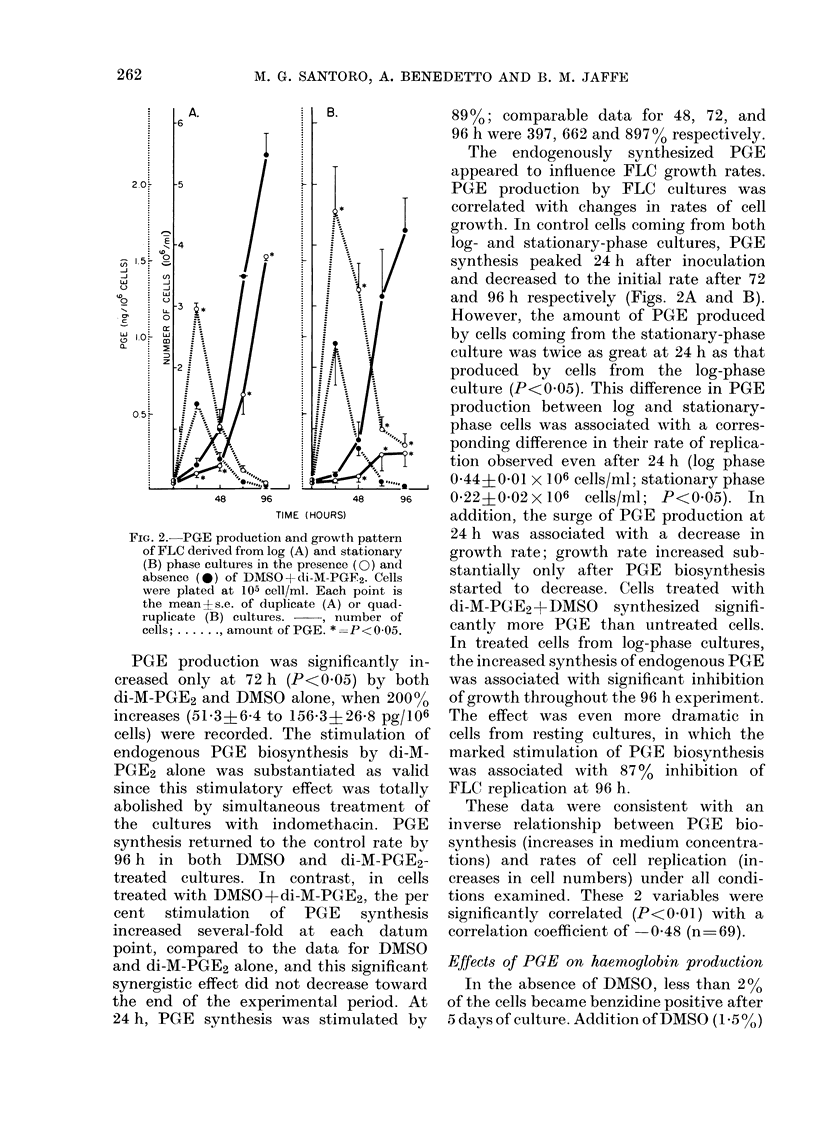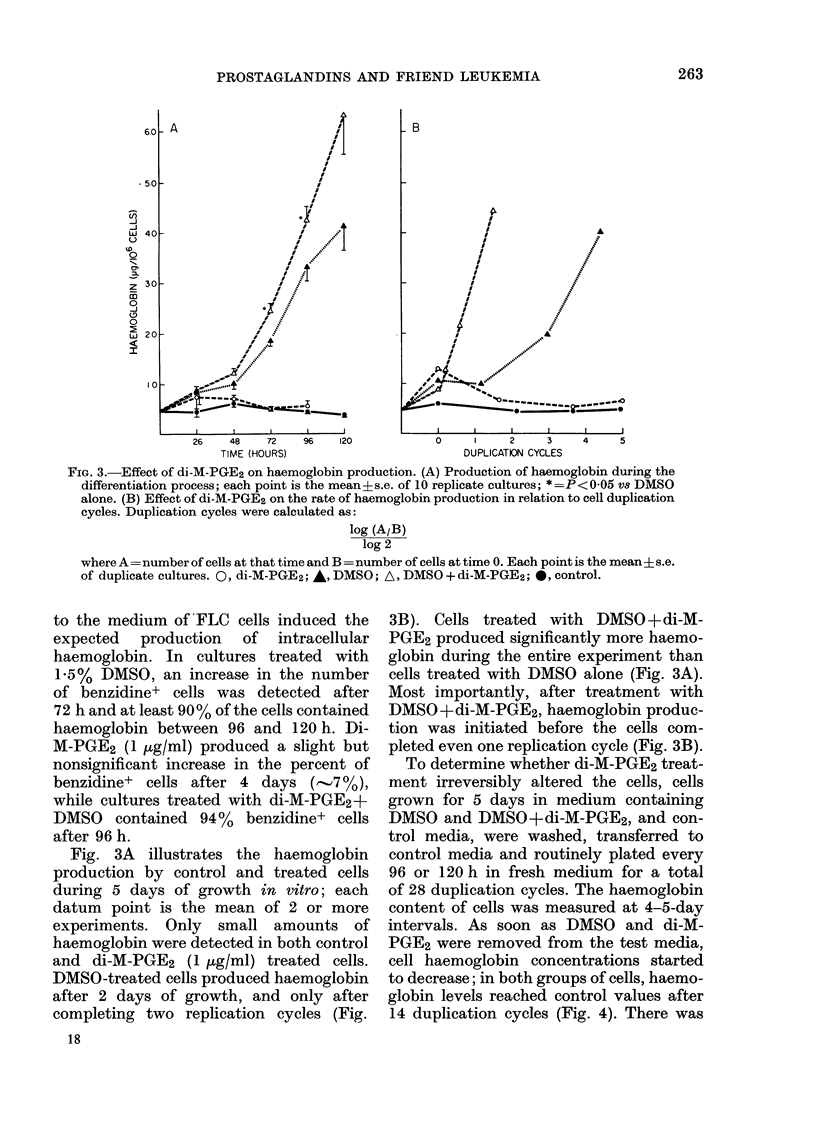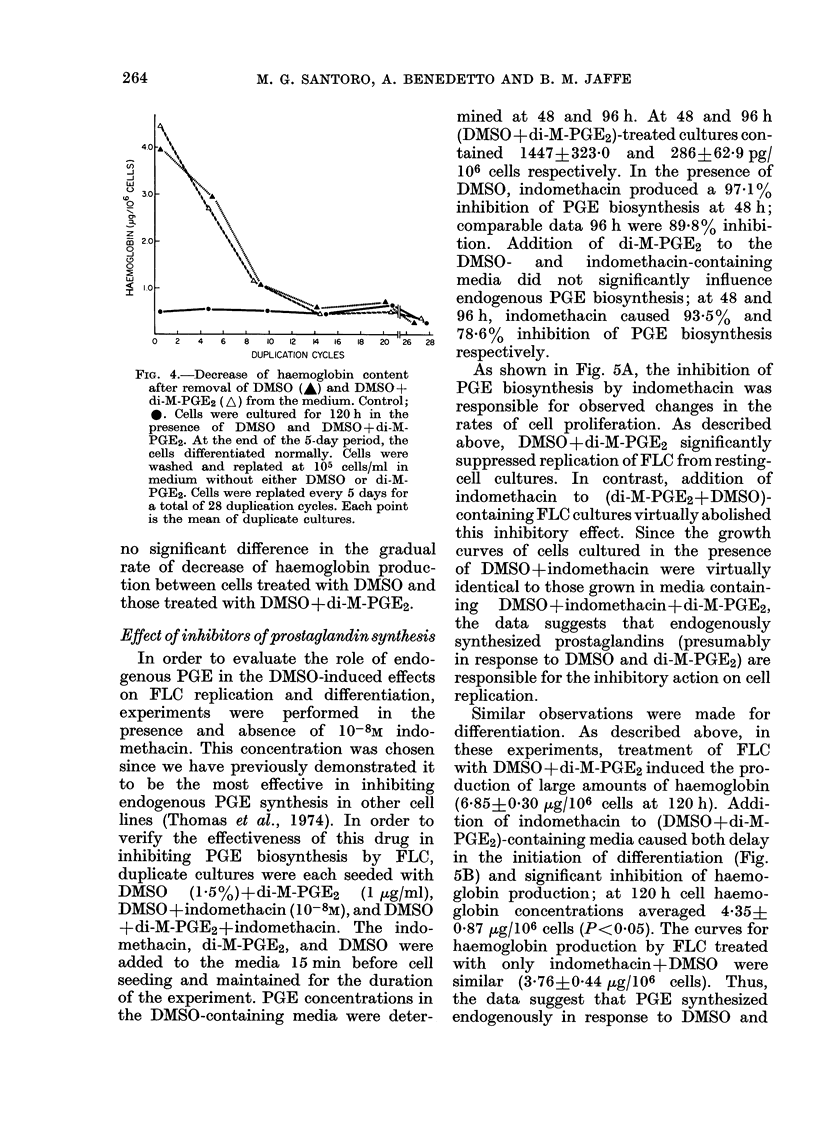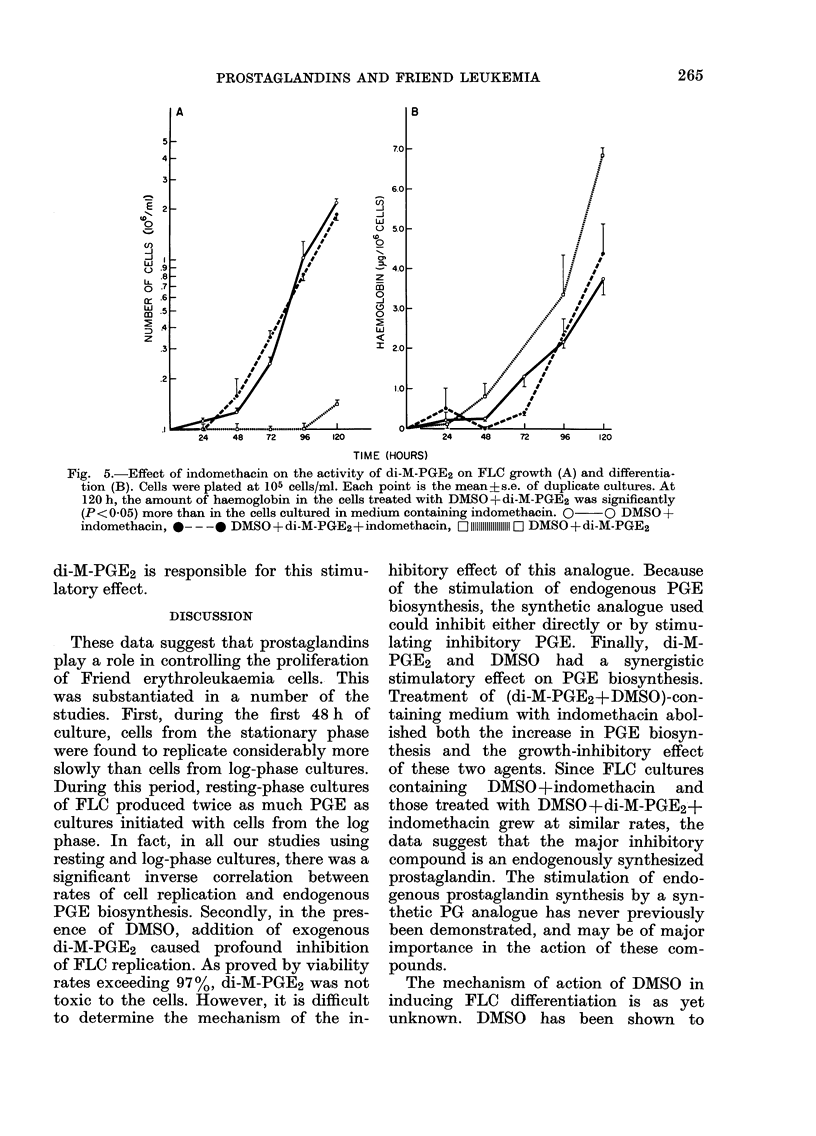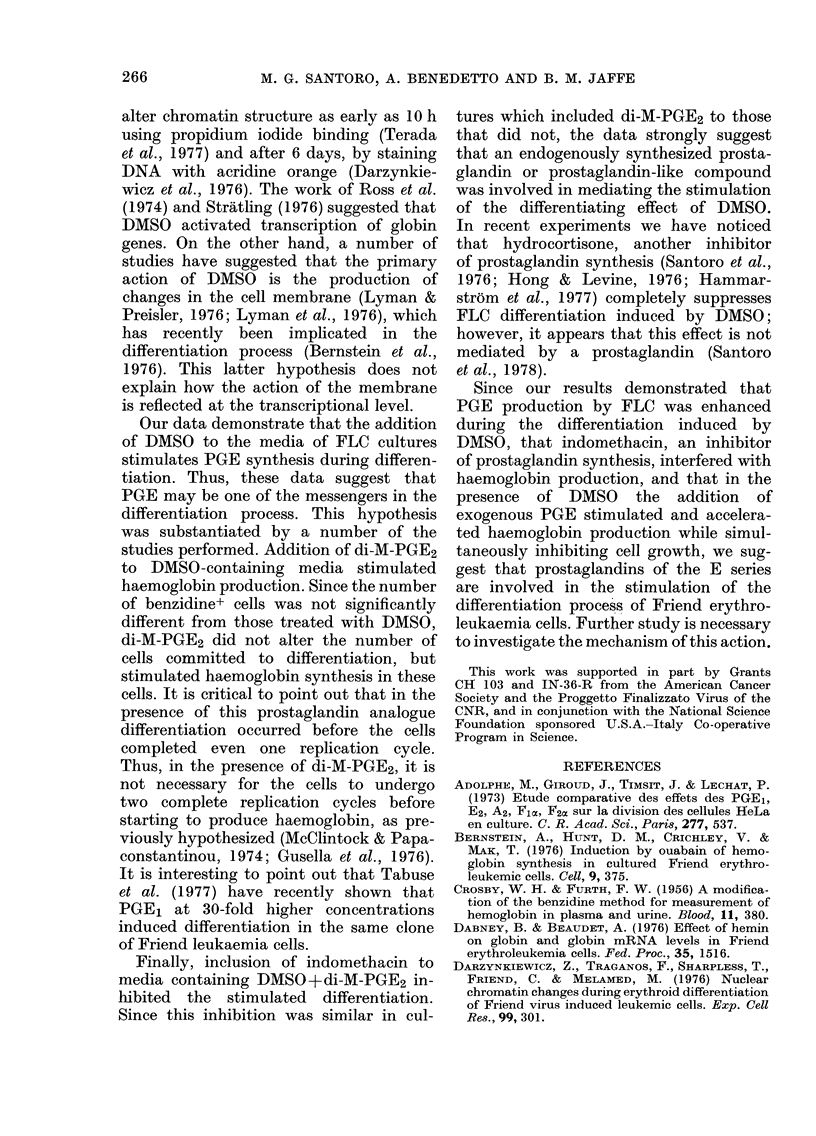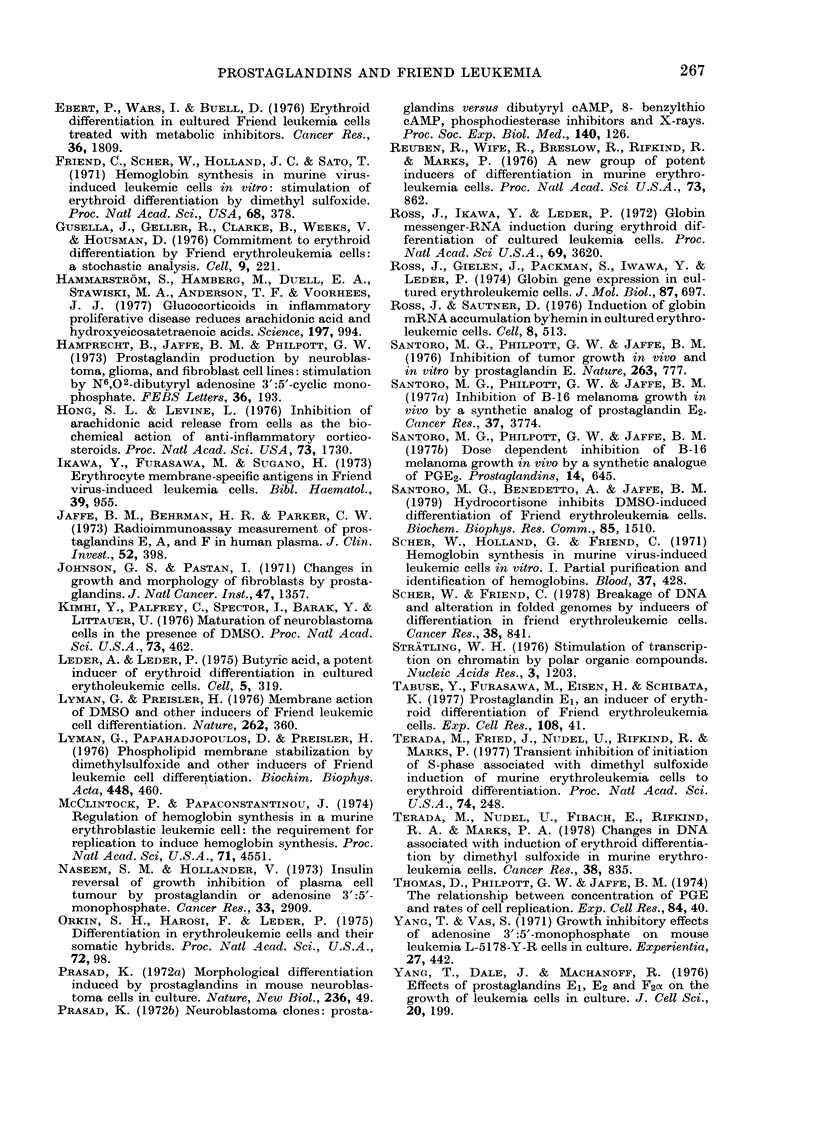# Effect of endogenous and exogenous prostaglandin E on Friend erythroleukaemia cell growth and differentiation.

**DOI:** 10.1038/bjc.1979.49

**Published:** 1979-03

**Authors:** M. G. Santoro, A. Benedetto, B. M. Jaffe

## Abstract

The effect of exogenous and endogenous prostaglandins on the patterns of growth and differentiation of Friend erythroleukaemia cells (FLC) were studied. During the differentiation process, DMSO stimulated PGE synthesis by an average of 95%. The addition of a long-acting synthetic analogue of PGE2,16,16-dimethyl-PGE2-methyl ester (di-M-PGE2) to the culture medium only slightly and temporarily slowed cell growth, with no appreciable induction of differentiation. However, in the presence of DMSO, the same concentration of di-M-PGE2 produced 73% inhibition of cell growth and accelerated and potently stimulated haemoglobin production. The action of both di-M-PGE2 and DMSO on cell proliferation was dependent upon the state of cell growth at the time of the administration of these compounds. FLC cultures treated with DMSO + di-M-PGE2 produced considerable amounts of haemoglobin before even one duplication cycle was completed. Both DMSO and di-M-PGE2 stimulated endogenous PGE biosynthesis, and the biosynthetic effect of these compounds was synergistic. Inhibition of endogenous prostaglandin synthesis by indomethacin completely abolished the effects produced by DMSO + di-M-PGE2 on the growth, and substantially reduced the stimulated differentiation of FLC. These data suggest that an endogenously synthesized prostaglandin plays a significant role in both the inhibition of replication and in the stimulation of differentiation induced by DMSO and di-M-PGE2 in Friend erythroleukaemia cells.


*Br. J. Cancer* (1979), **39**, 259

### EFFECT OF ENDOGENOUS AND EXOGENOUS PROSTAGLANDIN E ON FRIEND ERYTHROLEUKAEMIA CELL GROWTH AND DIFFERENTIATION

M. G. SANTORO,* A. BENEDETTO,† AND B. M. JAFFE

*From the *Department of Surgery, Washington University School of Medicine, St Louis, Missouri, 63310 and †Center of Virology, Ospedali Riuniti and CNR, Rome, Italy*

Received 6 October 1978 Accepted 4 December 1978

**Summary.—The effect of exogenous and endogenous prostaglandins on the patterns of growth and differentiation of Friend erythroleukaemia cells (FLC) were studied. During the differentiation process, DMSO stimulated PGE synthesis by an average of 95%. The addition of a long-acting synthetic analogue of $PGE_2$, 16,16-dimethyl-$PGE_2$-methyl ester (di-M-$PGE_2$) to the culture medium only slightly and temporarily slowed cell growth, with no appreciable induction of differentiation. However, in the presence of DMSO, the same concentration of di-M-$PGE_2$ produced 73% inhibition of cell growth and accelerated and potently stimulated haemoglobin production. The action of both di-M-$PGE_2$ and DMSO on cell proliferation was dependent upon the state of cell growth at the time of the administration of these compounds. FLC cultures treated with DMSO+di-M-$PGE_2$ produced considerable amounts of haemoglobin before even one duplication cycle was completed. Both DMSO and di-M-$PGE_2$ stimulated endogenous PGE biosynthesis, and the biosynthetic effect of these compounds was synergistic.**

**Inhibition of endogenous prostaglandin synthesis by indomethacin completely abolished the effects produced by DMSO+di-M-$PGE_2$ on the growth, and substantially reduced the stimulated differentiation of FLC. These data suggest that an endogenously synthesized prostaglandin plays a significant role in both the inhibition of replication and in the stimulation of differentiation induced by DMSO and di-M-$PGE_2$ in Friend erythroleukaemia cells.**

MURINE ERYTHROLEUKAEMIA CELLS infected with Friend virus (FLC) differentiate *in vitro* from a proerythroblast-like to a normoblast-like stage when treated with dimethylsulphoxide (DMSO) (Friend *et al.*, 1971) or other inducers (Leder & Leder, 1975; Reuben *et al.*, 1976; Ebert *et al.*, 1976; Dabney & Beaudet, 1976; Ross & Sautner, 1976). When induced to differentiate, FLC produce haemoglobin (Friend *et al.*, 1971) the electrophoretic pattern of which is similar to that of haemolysates of adult DBA/2j mice (Scher *et al.*, 1971), the strain from which the cells originated. Haemoglobin production is accompanied by morphological changes (Friend *et al.*, 1971), the appearance of erythrocyte membrane antigen (Ikawa *et al.*, 1973), an increase in iron uptake and haem synthesis (Friend *et al.*, 1971) and accumulation of mRNA for globin synthesis (Ross *et al.*, 1972; Ross *et al.*, 1974). It has been suggested that DMSO may act either directly on the genome of the cell (Ross *et al.*, 1972; Terada *et al.*, 1977; Darzynkiewicz *et al.*, 1976; Strätling, 1976; Terada *et al.*, 1978; Scher & Friend, 1978) or indirectly through an alteration in the cell membrane (Lyman & Preisler, 1976; Lyman *et al.*, 1976; Bernstein *et al.*, 1976) but the mechanism of action of DMSO on FLC is still unknown.

Address for correspondence: Bernard M. Jaffe, Department of Surgery, Washington University School of Medicine, 4960 Audubon Avenue, St Louis, Missouri 63110.

Prostaglandins of the E series have been shown to inhibit the growth of a number of tumour cell lines *in vitro*, including plasmacytoma (Naseem & Hollander, 1973), L-5178-Y-R mouse leukaemia (Yang & Vas, 1971; Yang *et al.*, 1976), HeLa, Hep-2, HT-29 (Adolphe *et al.*, 1973; Hamprecht *et al.*, 1973; Thomas *et al.*, 1974) and B16 melanoma (Santoro, Philpott & Jaffe, 1976; Santoro *et al.*, 1977*a*, 1977*b*). PGEs have also been shown to induce differentiation in mouse fibroblasts (Johnson & Pastan, 1971) and neuroblastoma cells (Prasad, 1972*a*, 1972*b*) as well as Friend leukaemia when used at very high concentrations (Tabuse *et al.*, 1977). The neuroblastoma cells have recently been shown to differentiate in the presence of DMSO (Kimhi *et al.*, 1976).

The purpose of the current study was to evaluate the effect of exogenous and endogenous prostaglandins on the pattern of growth and differentiation of Friend erythroleukaemia cells *in vitro*.

### MATERIALS AND METHODS

*Cells and culture techniques.*—Strain 745 (Cell Line GM-86) of Friend virus-induced erythroleukaemia cells of DBA/2j origin was provided by the Institute for Medical Research, Camden, N.J. The cells were grown in Dulbecco's modified Eagle's medium supplemented with 15% foetal calf serum, penicillin, 100 $\mu$/ml, and streptomycin, 0·1 mg/ml (GIBCO). Cultures were plated at a concentration of $10^5$ cells/ml and were maintained at 37±0·05°C in a humidified 95%-air, 5%-$CO_2$ atmosphere in 75-cm$^2$ plastic T-flasks (Falcon) containing 10 ml of media. Media were preincubated for 15–20 min before inoculation. 16, 16 dimethyl-$PGE_2$-methyl ester (di-M-$PGE_2$) was kindly provided by the Upjohn Co., Kalamazoo, Mich. It and indomethacin (Sigma) were dissolved in absolute ethanol and maintained at −20°C. Control media contained identical concentrations of ethanol (0·005%). Media containing di-M-$PGE_2$, indomethacin, DMSO (Fischer), or ethanol alone, were sterilized by Millipore filtration. At designated intervals, the number of cells in each of 2 or more replicate cultures was determined by counting in a haemocytometer; the standard error for replicate counts of the same cultures ranged from 2 to 6%. Cell viability determined by vital-dye exclusion (Trypan blue, 0·1%) ranged between 97 and 100% and was not influenced by the addition of di-M-$PGE_2$, indomethacin, DMSO, or ethanol at the concentrations used. Replication cycles were expressed as doubling in viable cell numbers. Resting-phase cultures were defined as containing cells cultured for 120–144 h without media changes and which, although viable for at least 96 h more, did not replicate further as they had reached saturation density.

*PGE assay.*—PGE concentrations in media and supernatants were measured by radioimmunoassay after organic solvent extraction and silicic acid chromatography as described previously (Jaffe *et al.*, 1973). Despite their intracellular action, immediately after they are synthesized prostaglandins are extruded from cells. Consequently, less than 10% of synthesized PGE is contained in the cells (Hamprecht *et al.*, 1973) and medium concentrations were measured as a way of quantifying the amounts of prostaglandin synthesized by cells. All prostaglandin determinations were corrected for appropriate medium blanks. The assay is quite specific; none of the compounds added to media, including di-M-$PGE_2$, cross-reacted with the anti-PGE antibody used.

Furthermore, PGF compounds, prostaglandin precursors and metabolites, thromboxane $B_2$, and 6-keto-$PGF_{1\alpha}$ (the inactive metabolite of prostacyclin) do not interfere with radioimmunoassay measurements of PGE.

*Benzidine staining.*—The cells (100 $\mu$l of cell cultures), prediluted to a concentration of $2\times10^5$ cells/ml, were stained by the addition of 50 $\mu$l of a solution containing 0·2% benzidine dihydrochloride, 0·75% hydrogen peroxide and 3% acetic acid (Orkin *et al.*, 1975) and benzidine-positive cells were counted by haemocytometer.

*Haemoglobin determination.*—Samples of cells ($2\times10^6$) were washed twice in sterile 0·9% NaCl and the pellets frozen at −70°C. Cells were lysed by repeated freeze-thawings (3×). Haemoglobin was measured from cell lysates using the technique of Crosby & Furth (1956).

*Statistics.*—Statistical comparisons were performed using a *t* test for unpaired data; *P* values less than 0·05 were considered significant.

RESULTS

*Effects of di-M-PGE$_2$ and DMSO on proliferation rates*

The effect of DMSO (1·5%) and DMSO +di-M-PGE$_2$ (1 $\mu$g/ml) on the growth of FLC is shown in Figs. 1A (cells from log-phase cultures) and B (cells from resting-phase cultures). At 24 h, di-M-PGE$_2$ alone (1 $\mu$g/ml) produced significant (18%) inhibition of growth, but at 48 h the inhibitory effect decreased and was not statistically significant (data not shown). The growth phase of the cells at the time of inoculation was shown to be an important variable. In cells from log-phase cultures, DMSO treatment did not significantly alter the growth pattern until 72 h, after which time (at 96 h) a 47% stimulation of cell growth ($P<0·05$) was noted. Treatment of these log-phase cells with DMSO+di-M-PGE$_2$ induced a mean growth inhibition of 45% for Days 1–3. After 96 h, the number of cells was not significantly different from the control cultures, but was significantly (42%) less than those in the DMSO-treated cells ($P<0·05$). In contrast, in cells from resting-phase cultures (Fig. 1B) DMSO caused significant inhibition of growth even after 72 h. Cells from resting-phase cultures were even more profoundly inhibited (73%) by treatment with DMSO+di-M-PGE$_2$ throughout the 5 days of culture.

*Effects of di-M-PGE$_2$ and DMSO on endogenous PGE biosynthesis*

The production of PGE during the growth of Friend leukaemia cells was determined. At the time of cell counts (every 24 h) aliquots of the supernatants of 4 replicate cultures from resting cell cultures and duplicate log-phase cultures were collected and assayed for PGE. Under control conditions, PGE biosynthesis reached a peak 24 h after inoculation, and decreased to the initial rate after 96 h (Figs. 2 A and B). The effect of addition to the medium of resting cell cultures of di-M-PGE$_2$ (1 $\mu$g/ml), DMSO (1·5%, v/v), and DMSO +di-M-PGE$_2$ on PGE biosynthesis was characterized.

FIG. 1.—Effect of DMSO (1·5%▲) and DMSO+di-M-PGE$_2$ (1 $\mu$g/ml △) on the pattern of growth of FLC derived from log (A) and stationary (B) phase cultures. Control (●). Cells were plated at 10$^5$ cell/ml. Di-M-PGE$_2$ and ethanol were added as described in Methods. Each point is the mean ± s.e. of data from duplicate (A)and 6 replicate (B) cultures. * = $P<0·05$ *vs* control, † = $P<0·05$ *vs* DMSO.

Fig. 2.—PGE production and growth pattern of FLC derived from log (A) and stationary (B) phase cultures in the presence (○) and absence (●) of DMSO+di-M-PGE2. Cells were plated at $10^5$ cell/ml. Each point is the mean±s.e. of duplicate (A) or quadruplicate (B) cultures. ——, number of cells; ......, amount of PGE. * = $P<0.05$.

PGE production was significantly increased only at 72 h ($P<0{\cdot}05$) by both di-M-$PGE_2$ and DMSO alone, when 200% increases (51·3±6·4 to 156·3±26·8 pg/$10^6$ cells) were recorded. The stimulation of endogenous PGE biosynthesis by di-M-$PGE_2$ alone was substantiated as valid since this stimulatory effect was totally abolished by simultaneous treatment of the cultures with indomethacin. PGE synthesis returned to the control rate by 96 h in both DMSO and di-M-$PGE_2$-treated cultures. In contrast, in cells treated with DMSO+di-M-$PGE_2$, the per cent stimulation of PGE synthesis increased several-fold at each datum point, compared to the data for DMSO and di-M-$PGE_2$ alone, and this significant synergistic effect did not decrease toward the end of the experimental period. At 24 h, PGE synthesis was stimulated by 89%; comparable data for 48, 72, and 96 h were 397, 662 and 897% respectively.

The endogenously synthesized PGE appeared to influence FLC growth rates. PGE production by FLC cultures was correlated with changes in rates of cell growth. In control cells coming from both log- and stationary-phase cultures, PGE synthesis peaked 24 h after inoculation and decreased to the initial rate after 72 and 96 h respectively (Figs. 2A and B). However, the amount of PGE produced by cells coming from the stationary-phase culture was twice as great at 24 h as that produced by cells from the log-phase culture ($P<0{\cdot}05$). This difference in PGE production between log and stationary-phase cells was associated with a corresponding difference in their rate of replication observed even after 24 h (log phase $0{\cdot}44\pm0{\cdot}01\times10^6$ cells/ml; stationary phase $0{\cdot}22\pm0{\cdot}02\times10^6$ cells/ml; $P<0{\cdot}05$). In addition, the surge of PGE production at 24 h was associated with a decrease in growth rate; growth rate increased substantially only after PGE biosynthesis started to decrease. Cells treated with di-M-$PGE_2$+DMSO synthesized significantly more PGE than untreated cells. In treated cells from log-phase cultures, the increased synthesis of endogenous PGE was associated with significant inhibition of growth throughout the 96 h experiment. The effect was even more dramatic in cells from resting cultures, in which the marked stimulation of PGE biosynthesis was associated with 87% inhibition of FLC replication at 96 h.

These data were consistent with an inverse relationship between PGE biosynthesis (increases in medium concentrations) and rates of cell replication (increases in cell numbers) under all conditions examined. These 2 variables were significantly correlated ($P<0{\cdot}01$) with a correlation coefficient of $-0{\cdot}48$ (n=69).

### *Effects of PGE on haemoglobin production*

In the absence of DMSO, less than 2% of the cells became benzidine positive after 5 days of culture. Addition of DMSO (1·5%)

FIG. 3.—Effect of di-M-PGE$_2$ on haemoglobin production. (A) Production of haemoglobin during the differentiation process; each point is the mean ± s.e. of 10 replicate cultures; * = $P < 0.05$ *vs* DMSO alone. (B) Effect of di-M-PGE$_2$ on the rate of haemoglobin production in relation to cell duplication cycles. Duplication cycles were calculated as:

$$\frac{\log (A/B)}{\log 2}$$

where A = number of cells at that time and B = number of cells at time 0. Each point is the mean ± s.e. of duplicate cultures. ○, di-M-PGE$_2$; ▲, DMSO; △, DMSO + di-M-PGE$_2$; ●, control.

to the medium of FLC cells induced the expected production of intracellular haemoglobin. In cultures treated with 1·5% DMSO, an increase in the number of benzidine$^+$ cells was detected after 72 h and at least 90% of the cells contained haemoglobin between 96 and 120 h. Di-M-PGE$_2$ (1 µg/ml) produced a slight but nonsignificant increase in the percent of benzidine$^+$ cells after 4 days (~7%), while cultures treated with di-M-PGE$_2$+DMSO contained 94% benzidine$^+$ cells after 96 h.

Fig. 3A illustrates the haemoglobin production by control and treated cells during 5 days of growth *in vitro*; each datum point is the mean of 2 or more experiments. Only small amounts of haemoglobin were detected in both control and di-M-PGE$_2$ (1 µg/ml) treated cells. DMSO-treated cells produced haemoglobin after 2 days of growth, and only after completing two replication cycles (Fig. 3B). Cells treated with DMSO+di-M-PGE$_2$ produced significantly more haemoglobin during the entire experiment than cells treated with DMSO alone (Fig. 3A). Most importantly, after treatment with DMSO+di-M-PGE$_2$, haemoglobin production was initiated before the cells completed even one replication cycle (Fig. 3B).

To determine whether di-M-PGE$_2$ treatment irreversibly altered the cells, cells grown for 5 days in medium containing DMSO and DMSO+di-M-PGE$_2$, and control media, were washed, transferred to control media and routinely plated every 96 or 120 h in fresh medium for a total of 28 duplication cycles. The haemoglobin content of cells was measured at 4–5-day intervals. As soon as DMSO and di-M-PGE$_2$ were removed from the test media, cell haemoglobin concentrations started to decrease; in both groups of cells, haemoglobin levels reached control values after 14 duplication cycles (Fig. 4). There was

FIG. 4.—Decrease of haemoglobin content after removal of DMSO (▲) and DMSO+di-M-$PGE_2$ (△) from the medium. Control; ●. Cells were cultured for 120 h in the presence of DMSO and DMSO+di-M-$PGE_2$. At the end of the 5-day period, the cells differentiated normally. Cells were washed and replated at $10^5$ cells/ml in medium without either DMSO or di-M-$PGE_2$. Cells were replated every 5 days for a total of 28 duplication cycles. Each point is the mean of duplicate cultures.

no significant difference in the gradual rate of decrease of haemoglobin production between cells treated with DMSO and those treated with DMSO+di-M-$PGE_2$.

*Effect of inhibitors of prostaglandin synthesis*

In order to evaluate the role of endogenous PGE in the DMSO-induced effects on FLC replication and differentiation, experiments were performed in the presence and absence of $10^{-8}$M indomethacin. This concentration was chosen since we have previously demonstrated it to be the most effective in inhibiting endogenous PGE synthesis in other cell lines (Thomas *et al.*, 1974). In order to verify the effectiveness of this drug in inhibiting PGE biosynthesis by FLC, duplicate cultures were each seeded with DMSO (1·5%)+di-M-$PGE_2$ (1 μg/ml), DMSO+indomethacin ($10^{-8}$M), and DMSO+di-M-$PGE_2$+indomethacin. The indomethacin, di-M-$PGE_2$, and DMSO were added to the media 15 min before cell seeding and maintained for the duration of the experiment. PGE concentrations in the DMSO-containing media were determined at 48 and 96 h. At 48 and 96 h (DMSO+di-M-$PGE_2$)-treated cultures contained 1447±323·0 and 286±62·9 pg/$10^6$ cells respectively. In the presence of DMSO, indomethacin produced a 97·1% inhibition of PGE biosynthesis at 48 h; comparable data 96 h were 89·8% inhibition. Addition of di-M-$PGE_2$ to the DMSO- and indomethacin-containing media did not significantly influence endogenous PGE biosynthesis; at 48 and 96 h, indomethacin caused 93·5% and 78·6% inhibition of PGE biosynthesis respectively.

As shown in Fig. 5A, the inhibition of PGE biosynthesis by indomethacin was responsible for observed changes in the rates of cell proliferation. As described above, DMSO+di-M-$PGE_2$ significantly suppressed replication of FLC from resting-cell cultures. In contrast, addition of indomethacin to (di-M-$PGE_2$+DMSO)-containing FLC cultures virtually abolished this inhibitory effect. Since the growth curves of cells cultured in the presence of DMSO+indomethacin were virtually identical to those grown in media containing DMSO+indomethacin+di-M-$PGE_2$, the data suggests that endogenously synthesized prostaglandins (presumably in response to DMSO and di-M-$PGE_2$) are responsible for the inhibitory action on cell replication.

Similar observations were made for differentiation. As described above, in these experiments, treatment of FLC with DMSO+di-M-$PGE_2$ induced the production of large amounts of haemoglobin (6·85±0·30 μg/$10^6$ cells at 120 h). Addition of indomethacin to (DMSO+di-M-$PGE_2$)-containing media caused both delay in the initiation of differentiation (Fig. 5B) and significant inhibition of haemoglobin production; at 120 h cell haemoglobin concentrations averaged 4·35±0·87 μg/$10^6$ cells ($P<0·05$). The curves for haemoglobin production by FLC treated with only indomethacin+DMSO were similar (3·76±0·44 μg/$10^6$ cells). Thus, the data suggest that PGE synthesized endogenously in response to DMSO and

Fig. 5.—Effect of indomethacin on the activity of di-M-PGE$_2$ on FLC growth (A) and differentiation (B). Cells were plated at $10^5$ cells/ml. Each point is the mean ± s.e. of duplicate cultures. At 120 h, the amount of haemoglobin in the cells treated with DMSO + di-M-PGE$_2$ was significantly ($P < 0 \cdot 05$) more than in the cells cultured in medium containing indomethacin. ○——○ DMSO + indomethacin, ●- - -● DMSO + di-M-PGE$_2$ + indomethacin, □|||||||||||||||□ DMSO + di-M-PGE$_2$

di-M-PGE$_2$ is responsible for this stimulatory effect.

### DISCUSSION

These data suggest that prostaglandins play a role in controlling the proliferation of Friend erythroleukaemia cells. This was substantiated in a number of the studies. First, during the first 48 h of culture, cells from the stationary phase were found to replicate considerably more slowly than cells from log-phase cultures. During this period, resting-phase cultures of FLC produced twice as much PGE as cultures initiated with cells from the log phase. In fact, in all our studies using resting and log-phase cultures, there was a significant inverse correlation between rates of cell replication and endogenous PGE biosynthesis. Secondly, in the presence of DMSO, addition of exogenous di-M-PGE$_2$ caused profound inhibition of FLC replication. As proved by viability rates exceeding 97%, di-M-PGE$_2$ was not toxic to the cells. However, it is difficult to determine the mechanism of the inhibitory effect of this analogue. Because of the stimulation of endogenous PGE biosynthesis, the synthetic analogue used could inhibit either directly or by stimulating inhibitory PGE. Finally, di-M-PGE$_2$ and DMSO had a synergistic stimulatory effect on PGE biosynthesis. Treatment of (di-M-PGE$_2$+DMSO)-containing medium with indomethacin abolished both the increase in PGE biosynthesis and the growth-inhibitory effect of these two agents. Since FLC cultures containing DMSO + indomethacin and those treated with DMSO + di-M-PGE$_2$ + indomethacin grew at similar rates, the data suggest that the major inhibitory compound is an endogenously synthesized prostaglandin. The stimulation of endogenous prostaglandin synthesis by a synthetic PG analogue has never previously been demonstrated, and may be of major importance in the action of these compounds.

The mechanism of action of DMSO in inducing FLC differentiation is as yet unknown. DMSO has been shown to alter chromatin structure as early as 10 h using propidium iodide binding (Terada *et al.*, 1977) and after 6 days, by staining DNA with acridine orange (Darzynkiewicz *et al.*, 1976). The work of Ross *et al.* (1974) and Strätling (1976) suggested that DMSO activated transcription of globin genes. On the other hand, a number of studies have suggested that the primary action of DMSO is the production of changes in the cell membrane (Lyman & Preisler, 1976; Lyman *et al.*, 1976), which has recently been implicated in the differentiation process (Bernstein *et al.*, 1976). This latter hypothesis does not explain how the action of the membrane is reflected at the transcriptional level.

Our data demonstrate that the addition of DMSO to the media of FLC cultures stimulates PGE synthesis during differentiation. Thus, these data suggest that PGE may be one of the messengers in the differentiation process. This hypothesis was substantiated by a number of the studies performed. Addition of di-M-$PGE_2$ to DMSO-containing media stimulated haemoglobin production. Since the number of benzidine$^+$ cells was not significantly different from those treated with DMSO, di-M-$PGE_2$ did not alter the number of cells committed to differentiation, but stimulated haemoglobin synthesis in these cells. It is critical to point out that in the presence of this prostaglandin analogue differentiation occurred before the cells completed even one replication cycle. Thus, in the presence of di-M-$PGE_2$, it is not necessary for the cells to undergo two complete replication cycles before starting to produce haemoglobin, as previously hypothesized (McClintock & Papaconstantinou, 1974; Gusella *et al.*, 1976). It is interesting to point out that Tabuse *et al.* (1977) have recently shown that $PGE_1$ at 30-fold higher concentrations induced differentiation in the same clone of Friend leukaemia cells.

Finally, inclusion of indomethacin to media containing DMSO+di-M-$PGE_2$ inhibited the stimulated differentiation. Since this inhibition was similar in cultures which included di-M-$PGE_2$ to those that did not, the data strongly suggest that an endogenously synthesized prostaglandin or prostaglandin-like compound was involved in mediating the stimulation of the differentiating effect of DMSO. In recent experiments we have noticed that hydrocortisone, another inhibitor of prostaglandin synthesis (Santoro *et al.*, 1976; Hong & Levine, 1976; Hammarström *et al.*, 1977) completely suppresses FLC differentiation induced by DMSO; however, it appears that this effect is not mediated by a prostaglandin (Santoro *et al.*, 1978).

Since our results demonstrated that PGE production by FLC was enhanced during the differentiation induced by DMSO, that indomethacin, an inhibitor of prostaglandin synthesis, interfered with haemoglobin production, and that in the presence of DMSO the addition of exogenous PGE stimulated and accelerated haemoglobin production while simultaneously inhibiting cell growth, we suggest that prostaglandins of the E series are involved in the stimulation of the differentiation process of Friend erythroleukaemia cells. Further study is necessary to investigate the mechanism of this action.

This work was supported in part by Grants CH 103 and IN-36-R from the American Cancer Society and the Proggetto Finalizzato Virus of the CNR, and in conjunction with the National Science Foundation sponsored U.S.A.–Italy Co-operative Program in Science.

### REFERENCES

ADOLPHE, M., GIROUD, J., TIMSIT, J. & LECHAT, P. (1973) Etude comparative des effets des $PGE_1$, $E_2$, $A_2$, $F_{1\alpha}$, $F_{2\alpha}$ sur la division des cellules HeLa en culture. *C. R. Acad. Sci., Paris*, **277**, 537.

BERNSTEIN, A., HUNT, D. M., CRICHLEY, V. & MAK, T. (1976) Induction by ouabain of hemoglobin synthesis in cultured Friend erythroleukemic cells. *Cell*, **9**, 375.

CROSBY, W. H. & FURTH, F. W. (1956) A modification of the benzidine method for measurement of hemoglobin in plasma and urine. *Blood*, **11**, 380.

DABNEY, B. & BEAUDET, A. (1976) Effect of hemin on globin and globin mRNA levels in Friend erythroleukemia cells. *Fed. Proc.*, **35**, 1516.

DARZYNKIEWICZ, Z., TRAGANOS, F., SHARPLESS, T., FRIEND, C. & MELAMED, M. (1976) Nuclear chromatin changes during erythroid differentiation of Friend virus induced leukemic cells. *Exp. Cell Res.*, **99**, 301.

EBERT, P., WARS, I. & BUELL, D. (1976) Erythroid differentiation in cultured Friend leukemia cells treated with metabolic inhibitors. *Cancer Res.*, **36**, 1809.

FRIEND, C., SCHER, W., HOLLAND, J. C. & SATO, T. (1971) Hemoglobin synthesis in murine virus-induced leukemic cells *in vitro*: stimulation of erythroid differentiation by dimethyl sulfoxide. *Proc. Natl Acad. Sci., USA*, **68**, 378.

GUSELLA, J., GELLER, R., CLARKE, B., WEEKS, V. & HOUSMAN, D. (1976) Commitment to erythroid differentiation by Friend erythroleukemia cells: a stochastic analysis. *Cell*, **9**, 221.

HAMMARSTRÖM, S., HAMBERG, M., DUELL, E. A., STAWISKI, M. A., ANDERSON, T. F. & VOORHEES, J. J. (1977) Glucocorticoids in inflammatory proliferative disease reduces arachidonic acid and hydroxyeicosatetraenoic acids. *Science*, **197**, 994.

HAMPRECHT, B., JAFFE, B. M. & PHILPOTT, G. W. (1973) Prostaglandin production by neuroblastoma, glioma, and fibroblast cell lines: stimulation by $N^6,O^2$-dibutyryl adenosine 3′:5′-cyclic monophosphate. *FEBS Letters*, **36**, 193.

HONG, S. L. & LEVINE, L. (1976) Inhibition of arachidonic acid release from cells as the biochemical action of anti-inflammatory corticosteroids. *Proc. Natl Acad. Sci. USA*, **73**, 1730.

IKAWA, Y., FURASAWA, M. & SUGANO, H. (1973) Erythrocyte membrane-specific antigens in Friend virus-induced leukemia cells. *Bibl. Haematol.*, **39**, 955.

JAFFE, B. M., BEHRMAN, H. R. & PARKER, C. W. (1973) Radioimmunoassay measurement of prostaglandins E, A, and F in human plasma. *J. Clin. Invest.*, **52**, 398.

JOHNSON, G. S. & PASTAN, I. (1971) Changes in growth and morphology of fibroblasts by prostaglandins. *J. Natl Cancer. Inst.*, **47**, 1357.

KIMHI, Y., PALFREY, C., SPECTOR, I., BARAK, Y. & LITTAUER, U. (1976) Maturation of neuroblastoma cells in the presence of DMSO. *Proc. Natl Acad. Sci. U.S.A.*, **73**, 462.

LEDER, A. & LEDER, P. (1975) Butyric acid, a potent inducer of erythroid differentiation in cultured erytholeukemic cells. *Cell*, **5**, 319.

LYMAN, G. & PREISLER, H. (1976) Membrane action of DMSO and other inducers of Friend leukemic cell differentiation. *Nature*, **262**, 360.

LYMAN, G., PAPAHADJOPOULOS, D. & PREISLER, H. (1976) Phospholipid membrane stabilization by dimethylsulfoxide and other inducers of Friend leukemic cell differentiation. *Biochim. Biophys. Acta*, **448**, 460.

MCCLINTOCK, P. & PAPACONSTANTINOU, J. (1974) Regulation of hemoglobin synthesis in a murine erythroblastic leukemic cell: the requirement for replication to induce hemoglobin synthesis. *Proc. Natl Acad. Sci, U.S.A.*, **71**, 4551.

NASEEM, S. M. & HOLLANDER, V. (1973) Insulin reversal of growth inhibition of plasma cell tumour by prostaglandin or adenosine 3′:5′-monophosphate. *Cancer Res.*, **33**, 2909.

ORKIN, S. H., HAROSI, F. & LEDER, P. (1975) Differentiation in erythroleukemic cells and their somatic hybrids. *Proc. Natl Acad. Sci., U.S.A.*, **72**, 98.

PRASAD, K. (1972*a*) Morphological differentiation induced by prostaglandins in mouse neuroblastoma cells in culture. *Nature, New Biol.*, **236**, 49.

PRASAD, K. (1972*b*) Neuroblastoma clones: prostaglandins *versus* dibutyryl cAMP, 8- benzylthio cAMP, phosphodiesterase inhibitors and X-rays. *Proc. Soc. Exp. Biol. Med.*, **140**, 126.

REUBEN, R., WIFE, R., BRESLOW, R., RIFKIND, R. & MARKS, P. (1976) A new group of potent inducers of differentiation in murine erythroleukemia cells. *Proc. Natl Acad. Sci U.S.A.*, **73**, 862.

ROSS, J., IKAWA, Y. & LEDER, P. (1972) Globin messenger-RNA induction during erythroid differentiation of cultured leukemia cells. *Proc. Natl Acad. Sci U.S.A.*, **69**, 3620.

ROSS, J., GIELEN, J., PACKMAN, S., IWAWA, Y. & LEDER, P. (1974) Globin gene expression in cultured erythroleukemic cells. *J. Mol. Biol.*, **87**, 697.

ROSS, J. & SAUTNER, D. (1976) Induction of globin mRNA accumulation by hemin in cultured erythroleukemic cells. *Cell*, **8**, 513.

SANTORO, M. G., PHILPOTT, G. W. & JAFFE, B. M. (1976) Inhibition of tumor growth *in vivo* and *in vitro* by prostaglandin E. *Nature*, **263**, 777.

SANTORO, M. G., PHILPOTT, G. W. & JAFFE, B. M. (1977*a*) Inhibition of B-16 melanoma growth *in vivo* by a synthetic analog of prostaglandin $E_2$. *Cancer Res.*, **37**, 3774.

SANTORO, M. G., PHILPOTT, G. W. & JAFFE, B. M. (1977*b*) Dose dependent inhibition of B-16 melanoma growth *in vivo* by a synthetic analogue of $PGE_2$. *Prostaglandins*, **14**, 645.

SANTORO, M. G., BENEDETTO, A. & JAFFE, B. M. (1979) Hydrocortisone inhibits DMSO-induced differentiation of Friend erythroleukemia cells. *Biochem. Biophys. Res. Comm.*, **85**, 1510.

SCHER, W., HOLLAND, G. & FRIEND, C. (1971) Hemoglobin synthesis in murine virus-induced leukemic cells *in vitro*. I. Partial purification and identification of hemoglobins. *Blood*, **37**, 428.

SCHER, W. & FRIEND, C. (1978) Breakage of DNA and alteration in folded genomes by inducers of differentiation in friend erythroleukemic cells. *Cancer Res.*, **38**, 841.

STRÄTLING, W. H. (1976) Stimulation of transcription on chromatin by polar organic compounds. *Nucleic Acids Res.*, **3**, 1203.

TABUSE, Y., FURASAWA, M., EISEN, H. & SCHIBATA, K. (1977) Prostaglandin $E_1$, an inducer of erythroid differentiation of Friend erythroleukemia cells. *Exp. Cell Res.*, **108**, 41.

TERADA, M., FRIED, J., NUDEL, U., RIFKIND, R. & MARKS, P. (1977) Transient inhibition of initiation of S-phase associated with dimethyl sulfoxide induction of murine erythroleukemia cells to erythroid differentiation. *Proc. Natl Acad. Sci. U.S.A.*, **74**, 248.

TERADA, M., NUDEL, U., FIBACH, E., RIFKIND, R. A. & MARKS, P. A. (1978) Changes in DNA associated with induction of erythroid differentiation by dimethyl sulfoxide in murine erythroleukemia cells. *Cancer Res.*, **38**, 835.

THOMAS, D., PHILPOTT, G. W. & JAFFE, B. M. (1974) The relationship between concentration of PGE and rates of cell replication. *Exp. Cell Res.*, **84**, 40.

YANG, T. & VAS, S. (1971) Growth inhibitory effects of adenosine 3′:5′-monophosphate on mouse leukemia L-5178-Y-R cells in culture. *Experientia*, **27**, 442.

YANG, T., DALE, J. & MACHANOFF, R. (1976) Effects of prostaglandins $E_1$, $E_2$ and $F_{2\alpha}$ on the growth of leukemia cells in culture. *J. Cell Sci.*, **20**, 199.